# “We have to clean ourselves to ensure that our children are healthy and beautiful”: findings from a qualitative assessment of a hand hygiene poster in rural Uganda

**DOI:** 10.1186/s12889-018-6343-3

**Published:** 2019-01-03

**Authors:** B. L. Harrison, C. Ogara, M. Gladstone, E. D. Carrol, J. Dusabe-Richards, A. Medina-Lara, J. Ditai, A. D. Weeks

**Affiliations:** 1 Sanyu Research Unit, Department of Women’s and Children’s Health, University of Liverpool, Liverpool, UK; 2 Sanyu Africa Research Institute (SAfRI), Mbale, Uganda; 3 Institute of Infection and Global Health, University of Liverpool, Mbale, Uganda; 4 Liverpool School of Tropical Medicine, Liverpool, UK; 5 University of Exeter, Exeter, UK; 6 Busitema University Faculty of Health Sciences, Tororo, Uganda

**Keywords:** Neonatal infection, Hand hygiene, Moments for hand hygiene poster, BabyGel, Health promotion, Poster

## Abstract

**Background:**

Neonatal sepsis is a major cause of mortality worldwide, with most deaths occurring in low-income countries. The World Health Organisation (WHO) ‘5 Moments for Hand Hygiene’ poster has been used to reduce hospital-acquired infections, but there is no similar tool to prevent community-acquired newborn infections in low-resource settings. This assessment, part of the BabyGel Pilot study, evaluated the acceptability of the ‘Newborn Moments for Hand Hygiene in the Home’ poster. This was an educational tool which aimed to remind mothers in rural Uganda to clean their hands to prevent neonatal infection.

**Methods:**

The BabyGel pilot was a cluster randomised trial that assessed the post-partum use of alcohol-based hand rub (ABHR) to prevent neonatal infections in Mbale, Uganda. Fifty-five women in 5 village clusters received the ABHR and used it from birth to 3 months postnatally, with use guided by the new poster. Following the study, 5 focus group discussions (FGDs) were conducted consisting of 6–8 purposively sampled participants from intervention villages. FGDs were audio-recorded, transcribed then translated into English. Transcripts were inductively coded using ATLAS.ti® and qualitatively analysed using thematic content analysis.

**Results:**

Most mothers reported that they understood the message in the poster (“The picture shows me you must use these drugs to keep your baby healthy”) and that they could adhere to the moments from the poster. Some participants used the information from the poster to encourage other caregivers to use the ABHR (“after explaining to them, they liked it”). Other potential moments for hand hygiene were introduced by participants, such as after tending to domestic animals and gardening.

**Conclusion:**

The poster was well-received, and participants reported compliance with the moments for hand hygiene (although the full body wipe of the baby has since been removed). The poster will be adapted into a sticker format on the ABHR bottle. More focus could be put into an education tool for other caregivers who wish to hold the baby. Overall, the study demonstrated the acceptability of an adapted version of the WHO Moments for Hand Hygiene poster in the introduction of an intervention in the community.

**Trial registration:**

ISRCTN67852437, registered 02/03/2015.

**Trial funding:**

Medical Research Council/ Wellcome Trust/ DfID (Global Health Trials Scheme).

## Background

Each year, 141,000 infants in Uganda die before reaching their fifth birthday, with one third of these deaths occurring in the neonatal period [1]. Although considerable progress has been made in the Millennium Development Goals by reducing child mortality rates by 42% in 16 years [2], approximately 39,000 neonatal deaths still occur every year [1] with sepsis being a major cause [3]. A lack of proper toilet hand-washing facilities [4] contributes to these high neonatal sepsis rates, as well as poor education about proper sanitation and hygiene amongst parents and other carers [5]. In the Ugandan National Household Survey 2012 [4], it was determined that 82% of households used toilets without hand washing facilities. Furthermore, recent pilot work from the BabyGel scoping survey established that 53% of mothers do not wash their hands regularly and 47% of mothers only wash their hands when they are heavily soiled [6].

**Fig. 1:**
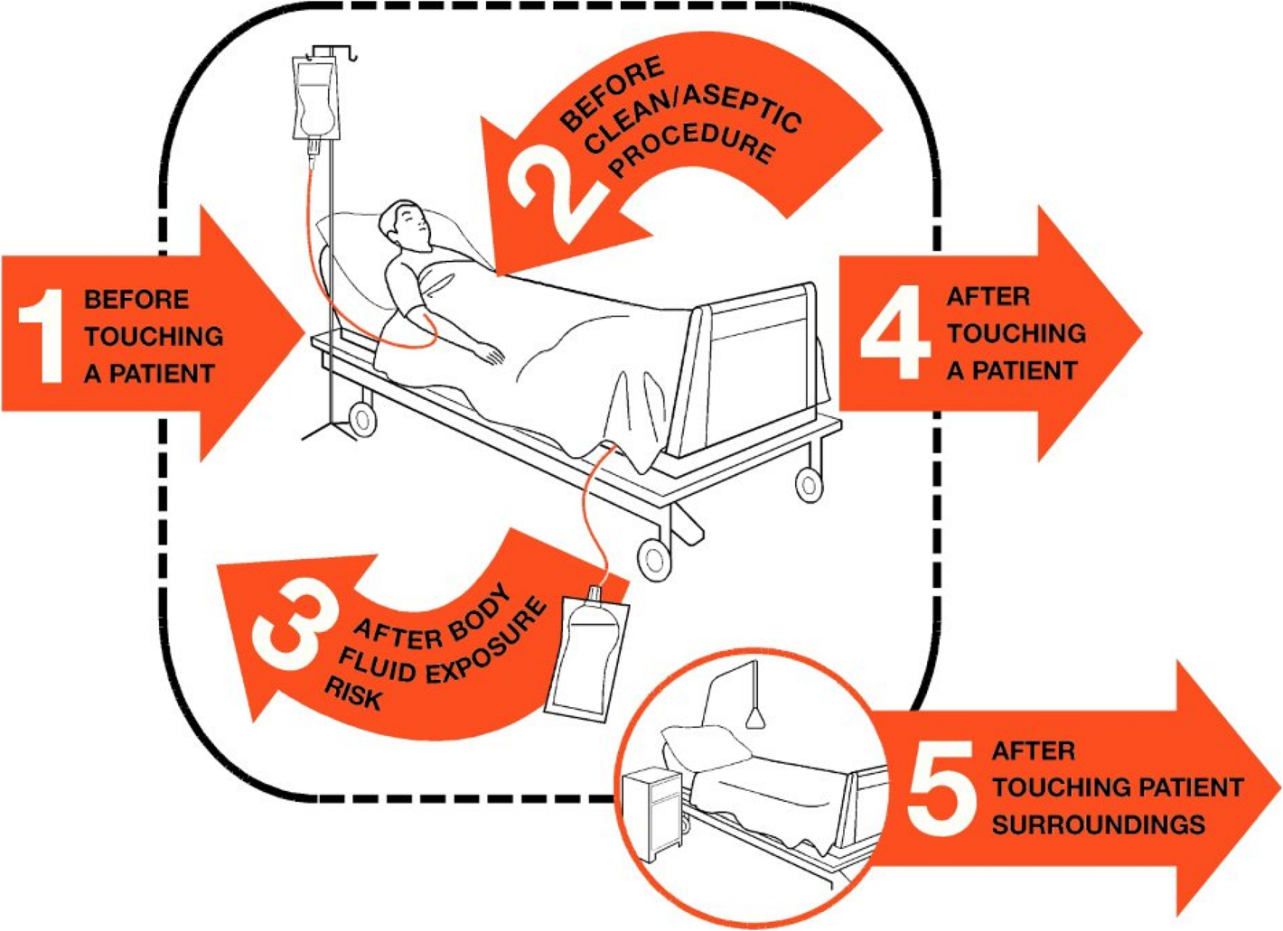
My Five Moments for Hand Hygiene

Hand hygiene is a high priority for the World Health Organisation (WHO), emphasised in their ‘Save Lives: Clean Your Hands’ global campaign [7]. The WHO ‘Five Moments for Hand Hygiene’ poster [8] (Fig. 1) provides advice on when it is most appropriate for healthcare professionals to wash their hands whilst caring for patients in a hospital setting. These five moments have been ratified as being effective by NICE [9]. The poster aims to avoid “misleading language and complicated descriptions” [10] and to standardise hand hygiene practices worldwide. However, as the poster’s target audience is hospital staff, its instructions rely on the availability of water and sanitation facilities. A problem arises when this is not available, as is the case in many rural Ugandan settings [4]. The WHO campaign does state that “hand rubs are not available or not affordable in many countries but … improving affordability and accessibility to this simple and proven intervention will save lives” [11]. If hand rubs were to be introduced, it is important that users are properly educated to ensure effectiveness of the intervention.

**Fig. 2:**
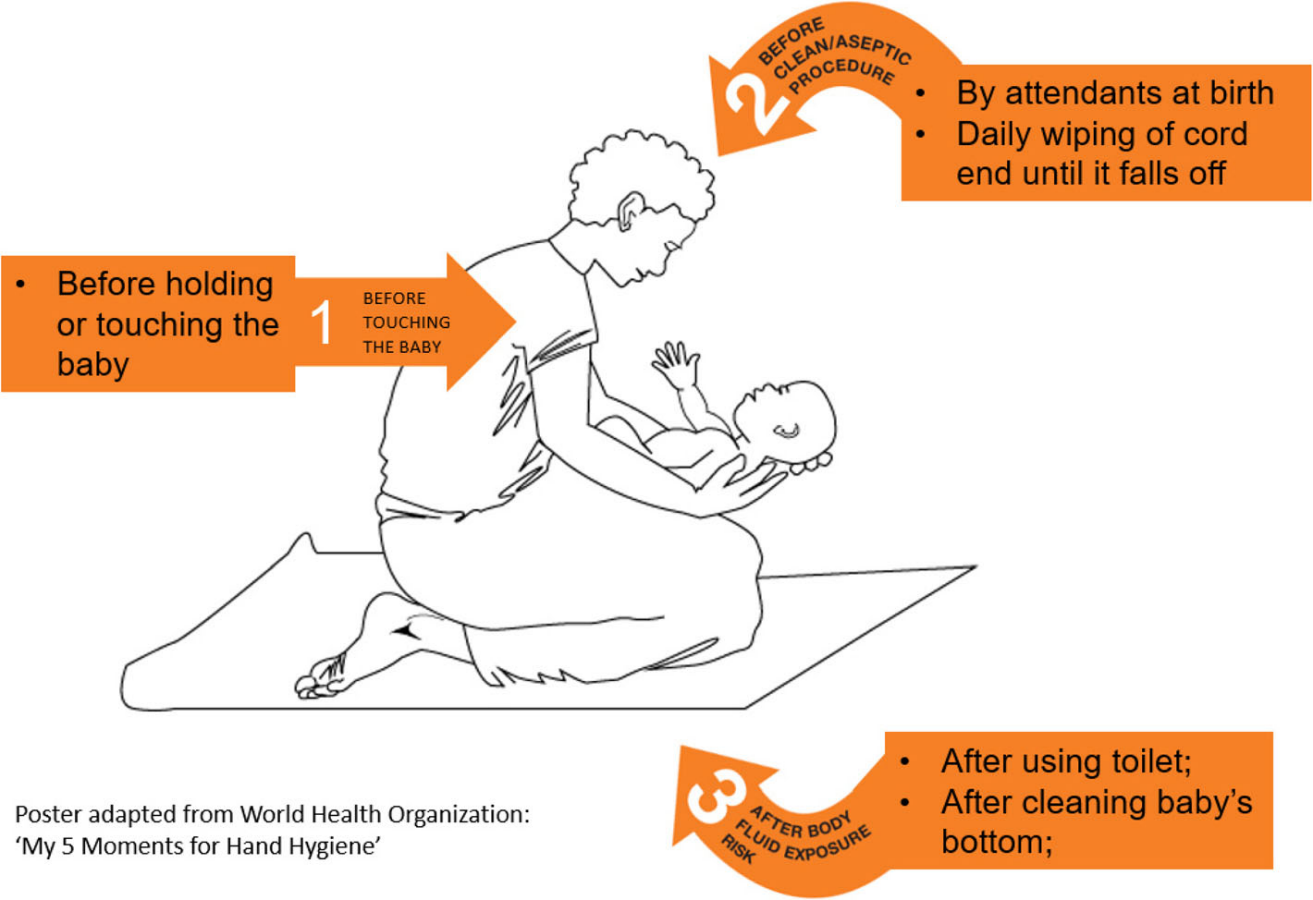
The ‘Newborn Moments for Hand Hygiene in the Home’ poster developed for the BabyGel study

The BabyGel study was a two-arm cluster randomised control trial to pilot the effectiveness of providing alcohol-based hand rub (ABHR) to mothers to prevent neonatal infection in the community. To educate mothers on when it is most appropriate to use the ABHR, the ‘Newborn Moments for Hand Hygiene in the Home’ poster was developed in collaboration with experts, based on WHO’s ‘5 Moments for Hand Hygiene’ campaign [12]. The poster has a simple illustration of a mother holding her baby, surrounded by the moments (Fig. 2). The number of moments were reduced from five to three. Moments 4 and 5, which relate to hand hygiene after touching the subject, were removed partly to simplify the image and partly because these moments are of only minor importance when caring for a single, healthy subject.

Posters are a low-cost method of health education, providing a visual and coherent portrayal of information. Despite posters frequently being used in African health promotion campaigns [13–15], there have been few studies evaluating their effectiveness or acceptability. Furthermore, there have been no studies in a rural African setting assessing the efficacy of educational posters to aid the use of interventions within clinical trials.

The World Health Organisation’s Action Plan is a commonly used guideline to implement the WHO multimodal hand hygiene improvement strategy [16]. This encourages training through multiple different approaches to optimise learning and compliance. Other interventions used for hand hygiene education have been in the form of lectures [17], education with fluorescent gel [18], and training sessions using mindfulness [19]. Overall, however, many hand hygiene campaigns achieve disappointing and unsustainable compliance [20]. Therefore, in this study, the poster was used as an adjunct to other hand hygiene education techniques – such as verbal teaching, support from village health workers and, later, the expert child.

The aim of this study was to determine whether this newly created poster with the 3 moments for hand hygiene was acceptable and understandable to parents in community settings in Uganda.

## Methods

### Setting

The BabyGel study is a cluster randomised control trial in Mbale District, Uganda, studying the effectiveness of alcohol hand gel in reducing neonatal infective morbidity. This is described in detail elsewhere (Ditai J, Weeks AD et al: BabyGel pilot: a pilot cluster randomised trial of the provision of alcohol hand gel to postpartum mothers to prevent neonatal and young infant infective morbidity in the community. In preparation). In an external pilot study, 10 villages were allocated to an intervention arm and a control arm, with eligible participants in the intervention arm receiving an ABHR antenatally, to use at the time of birth of the baby until the 90th day post-partum.

### Recruitment

A total of 55 pregnant women of over 34 weeks’ gestation were recruited to the intervention arm of the BabyGel study, from 5 villages. These villages were situated around Busiu Health Centre IV in Mbale District, Eastern Uganda and were selected to represent a variety of distances from each other, market areas and from the health centres. Participants were taught to use the hand rub at certain moments in their daily routine, as defined by the ‘Newborn Moments for Hand Hygiene’ poster. This was made available to all those in the intervention arm as a laminated colour poster in English or the dominant local language Lumasaba. The participants were taught verbally to use the ABHR at the moments specified in the poster and were supported by Village Health Workers.

At the end of the 3-month neonatal period, mothers from the intervention arm were invited to attend a focus group discussion (FGD) to offer their opinion on the acceptability and feasibility of the educational poster and ABHR. All 55 women in the BabyGel study intervention group were invited to participate, regardless of their level of education or literacy. A total of 35 women agreed to participate. Five focus groups were conducted throughout March and April 2016, each consisting of 6–8 participants, as summarised in Table 1.

**Table 1:** Demographics of FGD Participants

Village	Number of participants	Mean age of participant (range)
Namakye	8	22.6 (18–30)
Bulwalasi Toma	8	27.9 (19–37)
Namunyu	6	27.5 (19–39)
Makhonje 1	7	23.1 (19–30)
Makhonje 2	6	30.3 (19–36)

Most participants were married and described their occupation as a housewife or peasant farmer. Most had only primary education. The typical house was made from mud with an iron sheet roof. Most had non-ventilated pit latrines without handwashing facilities.

The FGDs were arranged by telephone call during the participants’ 90-day follow-up survey. On the day of the focus group, a research assistant from the Sanyu Africa Research Institute (SAfRI) formalised the participants’ consent prior to the discussion.

### Method of data collection

The FGDs were held in a convenient location, mainly in the church or home of a participant or village health worker. They were facilitated by SAfRI research assistants who are Ugandan scientists, nurses and psychologists who hold degrees and have previous experience in qualitative research. Their roles included taking notes, audio recording the session, and ensuring the discussion ran smoothly. One research assistant from each session acted as the moderator who asked questions from the pre-formulated topic guide and facilitated the running of the FGD.

**Fig. 3:**
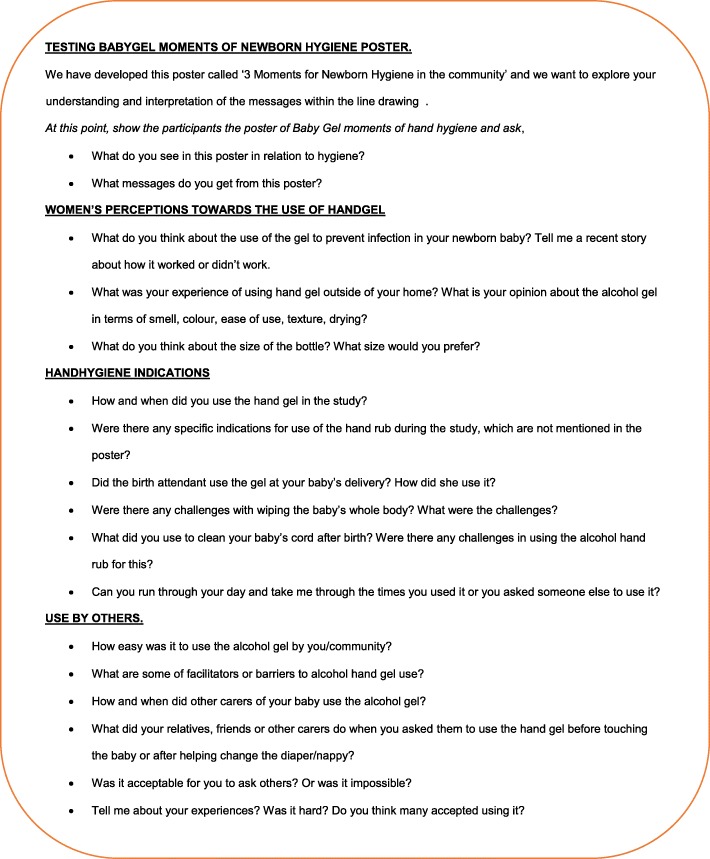
Topic guide for focus group discussions

A topic guide (Fig. 3) was developed, consisting of open questions formulated to explore participants’ answers in detail. As this was an iterative process, the topic guide was adapted after each focus group during team debrief sessions. This topic guide was used to direct the discussion of participants’ interpretation of the poster, suggestions of other moments for hand hygiene, and whether participants reported compliance with the moments for hand hygiene.

### Data analysis

Sessions were digitally recorded, with anonymous participants being identified only by their trial ID number. The recording was then transcribed by SAfRI research assistants, and quality-control checked by the senior research team in Mbale until they were satisfied that the grammar and terminology accurately reflected opinions of the participants. After transcription, the FGDs were then translated from Lumasaba into English using meaning-based translation. The translated script was then checked for legibility, accuracy and reproducibility by a fluent English speaker.

The transcript was qualitatively analysed using ATLAS.ti® software (Berlin, Germany) [21]. The transcripts were inductively coded line by line by BLH, using thematic content analysis whereby new codes were created for each new concept introduced by a participant. A table combining codes and quotations with interpretations of the data was created to condense the vast collection of quotes into the most common and important themes.

### Ethics

Ethical approval for the BabyGel pilot and associated nested studies was obtained before recruitment and was sought from the University of Liverpool Research Ethics Committee (RETH000808) and the Mbale Regional Referral Hospital Institutional Review Committee (HS1768). Funding for the project was from an MRC Development Grant.

## Results

The study findings demonstrated that most women had a good understanding of the poster, and subsequently reported good compliance with using the alcohol hand gel at the moments for hand hygiene outlined in the poster. The data were analysed thematically into 4 categories:

1. The participants’ interpretation of the poster
2. Reported compliance with the 3 moments for hand hygiene
3. Acceptability of other caregivers using the gel
4. Alternative moments for hand hygiene.

### Interpretation of the poster

Throughout the FGDs there were variable responses regarding participants’ interpretation of the poster: ranging from a literal interpretation, to having a high awareness for hand hygiene. Some mothers made the connection between the mother using the ABHR, being hygienic and therefore having a healthy and happy baby.

*“This picture shows me that in case you come from unclean environment, you must use these drugs to keep your baby healthy.”* FG1, participant B

*“I have seen a mother has smeared the child with this drug: the child is healthy.”* FG5, participant A

*“The message I get is that if you are from the toilet, you clean your hands before handling a baby.”* FG4, participant E

However, a small proportion of participants seemed to miss the concept of hygiene and only described the mother holding the baby. It also appears that participants only focused on the illustration within the poster, rather than the text surrounding it.

*“I have seen a mother carrying her child,”* FG3, participant C

*“I have seen a mother playing with a child.”* FG5, participant C

### Reported compliance with the 3 moments for hand hygiene

The first moment for hand hygiene involved using the ABHR before handling the baby. Examples of times when the alcohol hand gel was used included after housework, cooking or gardening, or before carrying the baby. All participants, with one exception, described that they followed the advice of this moment for hand hygiene.

*“You have to wash your hands using the gel before carrying the baby.”* FG1, participant H

*“When you’re from the garden or from the latrine, you have to wash your hands with it.”* FG3, participant D

Many participants accepted the importance of wiping the baby’s full body after birth, according to one of the three original moments for hand hygiene. Participants reported that their birth attendants used the gel during delivery, however sometimes the mother described wiping the baby herself or asking the midwife to use the ABHR as they were not aware of the BabyGel study.

*“She got a piece of cotton and made a drop of the drug on it to be able to start smearing the baby the whole body.”* FG1, participant E

The second moment for hand hygiene involved daily wiping of the umbilical cord with the alcohol gel until it dried or fell off. This moment was well-received with the participants – and many emphasised how well it worked by comparing it to experiences with their previous children. Women also described traditional cord-care practices, such as using salt and water, powder, and spirit. One participant described using lizard faeces to clean the cord, whilst another mother suggested using smoke residues.

*“My mother said that she was using the faeces of lizards … I told her that the nurse told me that this is the drug to be used and when I used it for only one week, it healed.”* FG4, participant G

*“My mother told me you use the smoke residues to smear the umbilical cord but this drug, in three days the umbilical cord had dried up and in a week it had healed”* FG4, participant B

Many women accepted the importance of using the gel after visiting the pit latrine, even if it was not used at any other time. Most women had a basic understanding that the toilet was home to many “germs.”

*“The message I get is that if you are from the toilet, you clean your hands before handling the baby.”* FG4, participant E

Participants seemed reluctant to discuss the use of the gel after infant anogenital care, with only 7 mothers mentioning this moment for hand hygiene.

*“The message I get is … after cleaning the baby who has defecated, you wash your hands before handling a baby.”* FG4, participant E

### Acceptability of other caregivers using the gel

Although the use of the alcohol hand gel by other caregivers was not an original moment for hand hygiene, many participants described their children, relatives, housekeepers and neighbours using the ABHR prior to carrying the baby. Most accounts of others using the hand gel were positive. Some participants described how they had to persuade others to use the ABHR, using their knowledge of how the alcohol hand gel works to encourage them.

*“I told them … the medical people are the ones who brought these drugs to help prevent infections in children. Later after explaining to them, they liked it.”* FG3, participant B

*“I tell her (the babysitter) first to wash her hands before she carries the baby.”* FG1, participant D

*“When they (the children) are playing outside, I tell them to first wash their hands with the gel before they go to play with the child.”* FG1, participant C

*“It’s good so I encourage others to use it because of its benefits.”* FG3, participant E

Most participants described that they had no difficulties in asking visitors and other caregivers to use the ABHR. However, for some it could lead to disagreements between the mother and visitors or relations. Although some reported initial challenges persuading other caregivers to use the gel, generally mothers had the confidence to describe the BabyGel pilot to people and encourage use of the ABHR.

*“I told them that once you refuse to wash your hands with the gel, I will never give you my child to carry.”* FG1, participant C

### Alternative moments for hand hygiene

The main alternative uses of the ABHR were the participants using the alcohol hand gel after tending to animals and after gardening. In rural Ugandan villages, many families keep domestic animals such as chickens and pigs, which can be a source of pathogens. Women also tend to fields of crops, and sometimes describe their hands being visibly soiled.

*“At home I have animals like pigs … I wash my hands using that drug before I handle my child.”* FG2, participant C

*“When you go to dig in the garden, you touch a lot of dirty things, so after the garden, you return and wash your hands with water and then you use the drug.”* FG4, participant G

Many participants felt that there were additional beneficial wound-healing properties of the gel.

*“In case there is a wound in the hand or something has scratched you, you can apply there that gel to make sure that the drug can help.”* FG1, participant D

*“His body parts had rashes so when I applied and used cotton, in two days, it had healed.”* FG4, participant D

Some women described using the ABHR prior to breastfeeding.

*“I use it in washing my hands before breastfeeding.”* FG1, participant D

Other alternative moments suggested by participants included; after housework, after cooking, or before greeting people.

*“After doing some domestic work like cooking, I smear it and when I go back I smear again.”* FG1, participant E

*“I use it a lot, from the toilet, after tying animals and when visitors come and I greet them.”* FG5, participant E

Occasionally it was reported that the gel was used inappropriately, such as children playing with the ABHR or some mothers describing how they used the gel remove stains from clothes.

*“Children tend to press it like this for fun … you have freedom that whenever it gets finished, you go and they refill, so that freedom is misused.”* FG2, participant C

*“We use it when we have wounds and stains on clothes, if you put it on stains and squeeze, it disappears.”* FG5, participant D

## Discussion

The three moments included in the poster for this study were well-received and understood by women in Ugandan communities, with participants reporting adherence to the moments for hand hygiene. The qualitative approach to this study with open-questioning in the FGDs allowed a detailed exploration of participants’ opinions and understanding of the poster. The iterative process of formulating the topic guide allowed some questions to be adjusted upon reflection.

In their interpretation of the poster, none of the mothers referenced the text surrounding the illustration; instead the focus was on the picture itself. This could be due to the word “poster” not translating properly into Lumasaba, and instead a word meaning ‘picture’ was used. It could also reflect illiteracy in the participants, as most participants were educated only to a primary level, therefore rendering the text of little use. Despite this, participants seemed to have a good understanding of the moments for hand hygiene, and this was reflected in their reported compliance throughout the study. This shows that the use of pictures can be a useful mechanism for portraying simple messages. However, it may be valuable to add a smaller picture to go with each moment to assist those who cannot read the text. This has been shown to be useful in a low-literacy population in Pakistan [22].

The full body wipe of the baby was included in the original ‘Moments for Hand Hygiene in the Home’ poster due to its effectiveness in pre-term babies in a randomised control trial in Nepal [23]. However, after it was developed, there was increasing concern that wiping the baby’s full body with alcohol would disrupt the normal floral of the skin and wipe away the vernix. The vernix contributes towards colonisation of normal bacterial flora in the gastrointestinal tract [24], and has been shown to be effective in developing the baby’s innate immune response [25]. Therefore, wiping away this natural substance could remove skin commensals, which would normally out-compete pathogenic bacteria. It was therefore decided that this moment would be removed from the ‘Moments for Hand Hygiene in the Home’ poster.

The second moment for hand hygiene involved cord care with the ABHR: this was a popular moment for hygiene, potentially because it had the most visible effects. Mothers discussed in detail the quick-healing properties of the alcohol gel, and how it replaced potentially dangerous health practices, such as smearing animal faeces on the cord. Such practices can become breeding grounds for a variety of pathogens and can be a severe risk to the baby’s health, causing omphalitis [26] and sepsis [27]. In low-resource settings, it is crucial that these local infections are prevented as identification of pathogens and prompt treatment is challenging: especially with growing antibiotic resistance. The rapid, visible, positive effects of cord care observed by mothers could reinforce the repeated use of ABHR and increase the probability of the woman using the gel again. This exhibits the theory of behaviourism [28] which describes how a learner’s actions are shaped through either positive or negative reinforcement.

Currently, the WHO guidelines [29] for Uganda recommend clean, dry cord care due to the country’s low mortality rate of under 30 neonatal deaths per 1000 live births [30]. However, in rural areas this mortality rate has been shown to be higher, up to 39 deaths per 1000 live births. Therefore, the most recent Uganda Clinical Guidelines suggest daily application of chlorhexidine on the cord stump until the cord falls off [31]. As omphalitis is a serious cause of neonatal sepsis, it is encouraging that participants reported compliance with this simple yet effective moment for hygiene and prefer the ABHR to traditional methods and dry cord care. This suggests that women will be happy to adopt modern cord care techniques, irrespective of whether this is ABHR or chlorhexidine.

Only a small number of participants described using the ABHR after infant anogenital toileting, which was unusual due to their reported adherence to all other moments for hand hygiene. However, there is a cultural taboo in Uganda regarding intimate anatomy such as genitalia, so participants could have adhered to advice of using the ABHR when changing the baby but preferred not to discuss it amongst others in the community. This could be a disadvantage of the focus group format, and one-to-one interviews could have removed this stigma from the conversation: however this would not have allowed the free-flowing discourse and interesting discussion that arose from the FGDs. It has also been argued [32] that sensitive or taboo topics are socially constructed and always changing, and focus groups can often allow participants to seek comfort and reassurance through discussion [33, 34]. It is therefore questionable whether this moment for hand hygiene was adhered to at all, and further research would need to be carried out to determine the cause of this potential non-adherence amongst participants.

Most participants were able to encourage other caregivers to use the alcohol hand rub at the specified moments for hand hygiene. However, some found it challenging, suggesting some kind of stigma surrounding the gel. This could have been due to it containing alcohol, or being cautious of its effectiveness in preventing infection. There have been concerns that ABHRs may be unacceptable to some religious groups [35], and in the setting where we conducted this study 15% of the population are Muslim. Although in Islamic law contact with alcohol is forbidden (Haram), guidance from international religious leaders have permitted its use [36, 37]. Locally, however, there may still be misconceptions, causing a barrier to ABHR use, even though this was not explicitly mentioned in the FGDs.

There also may have been concerns surrounding misuse of the ABHR. In another BabyGel nested study comparing the tolerance and acceptability of ABHR formulations [38], it was found that perfumed or bitterant additives were preferred over plain ABHR. The bitterant formulation was therefore chosen to be used in the Pilot to prevent misuse. WHO recommends that bitterants can be added to ABHR to prevent ingestion in high-risk areas – for example in paediatric settings or around patients with history of alcohol misuse [39].

On the other hand, many women in the FGDs described using their knowledge of the ABHR to encourage others to use the gel, showing that participant education can be crucial in compliance of an intervention. An educational tool could therefore be developed to explain to visitors and other caregivers what the BabyGel study is and when they should use the ABHR. This could allow participants to feel more comfortable when asking others to use the gel.

Another factor that could potentially encourage others to use the gel is the idea of learning through the observation of others. The social cognitive theory [40] describes the imitation of behaviour, reinforcing learning through personal, behavioural and environmental factors. If one member of the household, such as the main caregiver to the baby, uses the ABHR repetitively and likes it, this should prompt others to do the same.

Other external factors, separate from the results of the FGDs, also influenced the modification of the poster. One of these adjustments was regarding the accessibility of the poster. The poster was originally printed as an A4 laminated colour poster for participants to keep in their homes alongside the gel. In order to improve the accessibility of the poster, the image has since been developed into a sticker for the ABHR bottle. This enables the user to remind themselves of the moments for hand hygiene wherever they are and is more portable than the original A4 poster.

Another modification was the concept of the ‘expert child’. Before the FGDs occurred, it became evident that some participants were forgetting to use the ABHR. It was also noticed that most families had school-going children. We therefore identified these children and tasked them with reminding their mothers to use the ABHR before holding the baby. This was then developed into the concept of the ‘expert child,’ which will be incorporated further in the main BabyGel study (Ditai J, Weeks AD et al: BabyGel pilot: a pilot cluster randomised trial of the provision of alcohol hand gel to postpartum mothers to prevent neonatal and young infant infective morbidity in the community. In preparation), and used as an adjunct to the poster. There are few studies examining the bi-directional relationships between children and parents in the health and care setting, however some studies have shown that children could have a positive influence on a parent’s lifestyle and compliance with an intervention [41]. This idea could be taken forward and studied more closely in further research.

### Strengths and limitations

The authors are aware that there are limitations to this study. The relatively small sample size from a small Ugandan community may not be generalizable to other populations in Uganda or globally. It is also important to recognise that women with a higher understanding of the BabyGel study or the poster, or a higher education level may be more likely to engage with focus group discussions. There is also the possibility of bias when selecting quotes from the FGDs to use as examples in the Results section. The authors acknowledge this and chose the most thought-provoking quotes which most accurately represented common themes from the research findings.

Overall, this study has shown that the WHO “5 moments for hand hygiene” poster can be successfully adapted for different settings and populations – including newborn care in the community in a rural setting. This also fills the gap in the literature base evaluating a poster’s acceptability in a rural African setting and identifying whether it works to portray simple messages about health to aid an intervention in a randomised controlled trial.

## Conclusion

The ‘Moments for Hand Hygiene in the Home’ poster was well-received amongst participants in the intervention group of the BabyGel Pilot, with excellent reported compliance and understanding of the moments for hand hygiene. The poster will be adapted for the main study through results from the FGDs and other external factors. Firstly, the full body wipe of the baby will be removed from the poster due to its potential removal of the vernix, which has antimicrobial properties. As participants focussed largely on the illustration on the poster, appropriate individual pictures could be added to each moment for hand hygiene to increase acceptability to participants who have low reading skills. Finally, the poster will be adapted into a sticker format on the ABHR bottle to improve accessibility.

The poster has achieved its aims of being an acceptable education tool for teaching women in low-resource community settings when it is most appropriate to clean their hands, as it conveyed a strong yet simple message which overcame language and cultural barriers. However, the concept of ‘expert children’ could be used as an adjunct to the poster to remind participants to use the ABHR.

## Abbreviations

ABHR: Alcohol Based Hand Rub
FG (number): Focus Group (number)
FGD: Focus Group Discussions
NICE: The National Institute for Health and Care Excellence
SAfRI: Sanyu Africa Research Institute
WHO: World Health Organisation
